# The Importance of Quality Control of LSDV Live Attenuated Vaccines for Its Safe Application in the Field

**DOI:** 10.3390/vaccines9091019

**Published:** 2021-09-13

**Authors:** Andy Haegeman, Ilse De Leeuw, Meruyert Saduakassova, Willem Van Campe, Laetitia Aerts, Wannes Philips, Akhmetzhan Sultanov, Laurent Mostin, Kris De Clercq

**Affiliations:** 1 Unit of Exotic and Particular Diseases, Scientific Directorate Infectious Diseases in Animals, Sciensano, Groeselenberg 99, B-1180 Brussels, Belgium; Ilse.DeLeeuw@sciensano.be (I.D.L.); kris.declercq@sciensano.be (K.D.C.); 2 Kazakh Scientific Research Veterinary Institute (KazSRVI//KazNIVI), Raiymbek ave. 223, Almaty 050016, Kazakhstan; mika.kaznivi@gmail.com (M.S.); akhmetzhan.sultanov@gmail.com (A.S.); 3 Experimental Center Machelen, Scientific Directorate Infectious Diseases in Animals, Sciensano, Kerklaan 68, B-1830 Machelen, Belgium; Willem.VanCampe@sciensano.be (W.V.C.); Laurent.Mostin@sciensano.be (L.M.); 4 EURL for Diseases Caused by Capripox Viruses, Scientific Directorate Infectious Diseases in Animals, Sciensano, Groeselenberg 99, B-1180 Brussels, Belgium; Laetitia.Aerts@sciensano.be (L.A.); wannes.philips@sciensano.be (W.P.)

**Keywords:** vaccine, quality control, lumpy skin disease, recombinant

## Abstract

Vaccination is an effective approach to prevent, control and eradicate diseases, including lumpy skin disease (LSD). One of the measures to address farmer hesitation to vaccinate is guaranteeing the quality of vaccine batches. The purpose of this study was to demonstrate the importance of a quality procedure via the evaluation of the LSD vaccine, Lumpivax (Kevevapi). The initial PCR screening revealed the presence of wild type LSD virus (LSDV) and goatpox virus (GTPV), in addition to vaccine LSDV. New phylogenetic PCRs were developed to characterize in detail the genomic content and a vaccination/challenge trial was conducted to evaluate the impact on efficacy and diagnostics. The characterization confirmed the presence of LSDV wild-, vaccine- and GTPV-like sequences in the vaccine vial and also in samples taken from the vaccinated animals. The analysis was also suggestive for the presence of GTPV-LSDV (vaccine/wild) recombinants. In addition, the LSDV status of some of the animal samples was greatly influenced by the differentiating real-PCR used and could result in misinterpretation. Although the vaccine was clinically protective, the viral genomic content of the vaccine (being it multiple Capripox viruses and/or recombinants) and the impact on the diagnostics casts serious doubts of its use in the field.

## 1. Introduction

Lumpy skin disease virus (LSDV), causative agent of Lumpy skin disease (LSD), is a member of the genus *Capripox* (Capx) belonging to the family of *Poxviridae*. It is a double stranded DNA virus, approximately 150 kb long [1]. The virus was long contained to the African continent, but it has spread north- and eastwards, resulting in outbreaks in 2015–2016 in the Caucasus and the Balkan region, the Russian Federation and Kazakhstan [2], as well as the introduction of the disease on the Indian subcontinent [3] and several Southeast Asian countries (since 2019) [4]. The disease is typically characterized by the formation of nodules on the skin, not only causing painful lesions, but also permanent damage to the hide; it also causes also a drop in milk production, weight loss, abortion, infertility [5,6,7], and can even lead to death. Although mortality is general low [8], with outliers reaching up to 15% [9], and differences between European *Bos taurus* and *indicus* breeds [6], the disease does result in a significant loss of income, draught power, and fertilizers for agriculture [10,11]. Due to the significant socio-economic impacts [12], LSD has a notifiable status by the World Animal Health Organisation (OIE) and the European Union (Regulation EU 2016/429; Animal Health Law). As for any pathogen, human or animal, vaccination is a powerful tool to prevent, control, and eradicate a disease, especially combined with additional measures, such as movement restrictions. Vaccines come in a number of shapes, varying from killed, vectored and subunit vaccine to live attenuated vaccines (LAV). In combatting LSD, LAVs have been used most extensively as they were the only ones that were commercially available. The LAVs against LSDV can be further subdivided based upon the type of Capx virus used for attenuation, namely sheeppox virus (SPPV), goatpox virus (GTPV) or LSDV. The latter has been used with success in Israel in 2012–2013 [13], in the northern part of Cyprus in 2014–2015 [14] and in the Balkan region in 2016–2017 [15]. This observed protection efficacy in the field was confirmed by a recent comparison of several commercial LSDV-based LAVs under highly controlled and standardized laboratory setting [16]. In recent years, inactivated vaccines have gained attention in order to address the concerns about the side effects associated with the use of LSDV-based LAVs [13,17,18]. This resulted in the development of several inactivated vaccines and their potential as an alternative to the LAVs has been demonstrated in initial studies [18,19]. Independent of the vaccine properties, such as efficacy and safety, the use of vaccines has another requirement, namely production. A precise production process will vary from vaccine to vaccine and the overall process remains vulnerable to contaminates (chemical and/or biological) or fluctuation in a chemical/biological process resulting in titer differences. The introduction of a foreign agent can occur at different levels during the production process, such as the use of contaminated cell cultures or virus starting material, but also because of insufficient cleaning of materials/equipment or accidental re-use of non-sterilized material [20,21,22,23]. This becomes even more important when more than one vaccine is produced in parallel or in serial. Vaccination using a modified-live bluetongue virus vaccine contaminated with bovine viral diarrhea virus resulted in an outbreak in a herd of 28 Rocky Mountain bighorn sheep in Colorado [24]. Another example can be found in the contamination of the Newcastle disease virus (NDV)-attenuated vaccine with fowl adenovirus type 4 (FAdV-4). The synergistic reaction of FAdV and NDV resulted in significant increase of mortality in vaccinated chickens [25]. Bluetongue virus (BTV) has been isolated from commercial sheeppox and LSDV vaccines [26] which has potential detrimental consequences as sheep and cattle are both susceptible hosts for BTV. Not well enough attenuated BTV-2 modified live vaccine viruses was the causative agents of abortion in livestock in Italy [27] and foot-and-mouth disease serotype C outbreaks that occurred in Kenya between 1992 and 2004 were most probably related to vaccine escapes [28]. The few presented examples clearly demonstrate the necessity of a continued vigilance notwithstanding the improved production procedures.

It is the purpose of this study to demonstrate the need of independent vaccine quality control by presenting the data from a quality assessment study of a commercial LSDV-based live attenuated vaccine.

## 2. Materials and Methods

### 2.1. Cell Line, Challenge Virus and Vaccine 

The ovine testis cell line OA3.Ts cells (ATCC-CRL-6546) were cultured in DMEM (Thermo Fisher Scientific, Merelbeke, Belgium) supplemented with 10% fetal calf serum (FCS; Thermo Fisher Scientific, Merelbeke, Belgium), fungizone (1 µg/mL; Thermo Fisher Scientific, Merelbeke, Belgium) and gentamycin (20 µg/mL; Thermo Fisher Scientific, Merelbeke, Belgium). The LSDV strain LSD/OA3-Ts.MORAN was used as the challenge strain and kindly provided by the Kimron Veterinary Institute, Israel and the Israeli Veterinary Services, was propagated on OA3.Ts., as described by Haegeman et al. (2021) [16]. The following vaccines were used for this study: (i) Lumpivax (Kenya Veterinary Vaccines Production Institute, KEVEVAPI; Nairobi, Kenya; 100Doses batch NO:02/019); (ii) Lumpy Skin Disease Vaccine (abbreviated for this study as OBP-vac) (Onderstepoort Biological Products, OBP; Onderstepoort, South-Africa; batch 473); and (iii) Caprivac vaccine (Jordan Bioindustries Center, Jovac; Amman, Jordan; batch 210115-01).

### 2.2. Vaccine Efficacy Animal Trial Design

The efficacy of the Lumpivax was analyzed in accordance with the OIE Manual Chapter 3.4.12 Section 2.2.4 [29] and with our vaccine evaluation studies, described previously [16]. All animals (*n* = 12) were approximately 6-month-old, male Holstein bulls, which were tested free of BTV, BVD and IBR. Upon arrival, all animals were acclimated for 5 to 7 days to reduce the impact of the transport (stress) on the health parameters of the animals [30]. After an acclimatization period, 7 out of 12 animals were vaccinated (R1V to R7V) according to the manufacturer’s instructions, while 5 other animals remained untreated (challenge control animals; R1_con to R5_con). At 21 days post-vaccination (dpv) the vaccinated as well as the non-vaccinated control animals were challenged [16] with a virulent Israeli LSDV field strain (LSD/OA3-Ts.MORAN; titer 7.5–8 TCID50/mL) by intravenous (5 mL, vena jugularis) and intradermal (1 mL) inoculation. The intradermal injection was performed at 2 locations on both sides of the neck (250 µL per site). After the challenge the animals were monitored and sampled for at least 21 days.

The animal experiment was conducted according to the European Union and Belgian regulations on animal welfare in experimentation. The protocol was approved by the joined Ethical Committee of Sciensano, authorization number 20200302-01.

### 2.3. Clinical Evaluation, Scoring and Sampling

During the complete duration of the animal trial (acclimatization, post-vaccination and post-infection) all animals were daily clinically evaluated and scored, as described in Haegeman et al. (2021) [16].

Samples (EDTA and clotted blood, buccal swabs) for laboratory evaluation were taken as follows: (1) once during the acclimatization period; (2) on the day of vaccination but before the injection (0 dpv); (3) twice a week during the post-vaccination period; (4) on the day of challenge but before the injection (0 dpi); (5) every week day from 6 to 14 dpi and every other day before and after this period. Biopsies were taken when nodules first appeared to check for the presence of LSDV. At necropsy, approximately 25 to 26 tissue and organ samples were collected per vaccinated animal, similar to Haegeman at al. (2021) [16].

### 2.4. Viral DNA Extraction

Viral DNA from blood (200 µL), tissue and organ samples (25 mg) was extracted using the NucleoSpin Blood and NucleoSpin tissue kits (Macherey-Nagel, Düren, Germany), as described by the manufacturer with the exception of: (a) the incubation time for lysis of blood samples was prolonged to 1 h; (b) an external control (EC) was added to B3 buffer before extraction [31]; (c) tissue/organ samples were homogenized using a TissueLyser (Qiagen, Gaithersburg, MD, USA) in the presence of buffer T1 and proteinase K before the overnight incubation at 56 °C. Viral DNA from swabs was extracted by placing them into 1 mL PBS for 15 min at room temperature. Subsequently, the swabs were vortexed for 1 min and the DNA was extracted using the NucleoSpin Blood kit, as described above.

### 2.5. Virology

#### 2.5.1. Pan Capripox Real-Time PCR

Lumpy skin disease virus genome was detected using the panCapx real-time PCR panel, consisting of three real-time PCRs (D5r, E3L and J6R) each with an internal and external control as described by Haegeman et al. (2013) [32]. The panel was used as follows: after an initial screening with the D5R real-time PCR, samples were tested with the E3L and J6R real-time PCRs if: (a) the Cp > 37 or (b) in a time consecutive sample series the status of the animal changed (negative to positive and vice versa).

#### 2.5.2. Assays for Differentiating Infected from Vaccinated Animals (DIVA)

Differentiation between LSDV vaccine and wild type LSDV genome was performed using the real-time DIVA PCR’s described by Agianniotaki et al. (2017) [33] and Vidanović et al. (2021) [34]. These are referred to in the text as DIVA-1 and DIVA-2, respectively. The DIVA gel-based PCR described by Chibssa et al. (2018) was utilized for within Capx differentiation [35] (DIVA-3).

#### 2.5.3. Phylogenetic PCRs

In addition to the phylogenetic PCRs published by Lamien et al. (2013) [36] and Haegeman et al. (2016) [37], amplifying parts of RPO30 gene and ORF25/26 respectively, 4 new PCRs were developed in order to investigate sequence differences. Primer design was based upon the alignment of publicly available Capx genome sequences (NCBI nucleotide database: http://www.ncbi.nlm.nih.gov/ accessed on 1 October 2020) using the primer 3 software [38]. All Primers were designed in regions conserved among Capx viruses allowing them to be used for LSDV as well as SPPV and GTPV. The fragment size was kept around 700 bp for each amplicon and the cycling profile was kept identical to one of the published PCR [37]. Primer sequence information, fragment length and targeted region are summarized in Supplementary Table S1. The following reaction mix and cycling profiles were used for all 4 new PCRs: (1) the total reaction volume was 50 µL and consisted of 2 µL Capx DNA, 5 µL PCR Taq buffer (10×), 2.5 mM MgCl_2_, 0.3 mM of each dNTP (Roche Applied Science, Vilvoorde, Belgium), 1.25 U Taq Platinum Polymerase (Life Technologies, Gent, Belgium) and 33.75 pmol of a forward and reverse primer; (2) the thermal cycling profile was one cycle of 95 °C for 4 min; 45 cycles of 95 °C 30 s, 57 °C 30 s, 72 °C 1 min; one cycle of 72 °C for 10 min.

### 2.6. Serology

Sera samples were analyzed using the immunoperoxidase monolayer assay (IPMA) as described in Haegeman et al. (2020) [39] and the ID Screen^®^ Capripox Double Antigen Multi-species ELISA (Innovative Diagnostics, Grabels, France) according to manufacturer’s instructions. 

### 2.7. IFNg Release Assay

The secretion of interferon gamma (IFNg), as a measure of the cellular immune response of the host, was examined for all vaccinated animals as described in detail in Haegeman et al. (2021) [16]. In brief: following the stimulation of heparinized blood with either LSDV, PBS (negative control) or pokeweed mitogen (positive control) the secretion of IFNg was examined using the sandwich ELISA BOVIGAM^®^ 2G (Thermo Fisher Scientific, Merelbeke, Belgium). The cut-off for positivity was set at 0.3. The OD values of the positive and negative controls were monitored over time to identify false positive and negative results. The corrected response, ODcorrected = OD virus − OD PBS, was classified as strong (ODcorrected > 2), medium (ODcorrected between 1 and 2) or weak (ODcorrected < 1).

### 2.8. Cloning, Purification and Sanger Sequencing

After gel electrophoresis, the desired bands were excised and the corresponding PCR fragments were purified using the QIAquick Gel Extraction Kit (Qiagen, Venlo, the Netherlands). A total of 4 µL of each purified PCR fragment was ligated into the pCR2.1-Topo vector using the TOPO TA Cloning system (Invitrogen, Merelbeke, Belgium). Following blue/white screening on X-gal containing kanamycin (50 µg/mL) Luria-Bertani (LB) broth plates, plasmids were purified using the GeneJET Plasmid Miniprep Kit (Thermo Fisher Scientific, Merelbeke, Belgium) according to manufacturer’s instructions. Insert verification was carried out by Eco RI digestion and gel electrophoresis. The identity of the plasmids or purified PCR fragments was determined by sanger sequencing. The quality and consensus assembly was carried out using a combination of BlastN search and contig assembly in Genedoc [40].

### 2.9. Analysis of Potential Recombination Events

Aside the obtained sequences, a relevant dataset was generated for the Lumpivax region 4 by using in-house sequence data or from GenBank. In total there were 7 GTPV, 6 SPPV (4 vaccines) and 7 LSDV (4 vaccines) sequences included (Supplementary Table S2). For the analysis of the OBP/Caprivac mixture only the parental sequences were included. Potential recombination events were analyzed using the RDP4 software package (version 4.101) [41]. All seven methods available in the RDP4 were used, including RDP [42], GENECONV [43], Bootscan/Recscan method [44], 3Seq [45], Chimaera [46], SiScan [47] and MaxChi [48]. Defaults setting were applied including the *p*-value cut-off of 0.05 for detecting true recombinants.

## 3. Results

### 3.1. Identity Control of Vaccine Strains

Following reconstitution of the vaccines as recommended by each manufacturer, the identity of viral genomes present was examined. The three vaccines tested positive in the panCapx real-time PCR with Cp-values of 23.7 for OBP-vac, 28.5 for Lumpivax, and 22.3 for Caprivac. For the OBP-vac, the vaccine status was confirmed as only a signal (Cp 23.5) was obtained in the vaccine channel of the DIVA-1 and DIVA-2 real-time PCRs and LSDV status was confirmed by the DIVA-3 as the amplified fragment migrated similar to the LSDV reference sample after gel electrophoresis and no trace of a GPV/SPPV-band was observed. In contrast, for the Lumpivax a signal, in the DIVA-1 real-time PCR, was obtained in the vaccine as well as in the wild type channel, with a Cp-value of 29.6 for both. Electrophoretic analysis of the DIVA-3 PCR revealed not only an amplicon that migrated similarly to the LSDV reference sample, but also a second one at the same level as non-RM65 SPPV and GPTV controls. The result of the DIVA-1 real-time PCR for Lumpivax was confirmed by the DIVA-2 real-time PCR as also here a signal was obtained in the vaccine (Cp 22.8) and in the wild type channel (Cp 22.5). For the Caprivac vaccine, no LSDV (wild or vaccine type) was detected in the vaccine vial using DIVA-2. The GTPV status was further confirmed by DIVA-3 in combination with cloning. Six clones were randomly selected and sequenced. All clones were very similar (0 to 2 nt differences) and showed the highest identity with GTPV Gorgan (accession number: KX576657; 100 to 99.21%) [49].

### 3.2. Additional Characterization of the Lumpivax Vaccine

In order to characterize further the viral genome(s) present in the Lumpivax vaccine, it was decided to amplify six genomic regions spread across the Capx genome (Supplementary Table S1). The amplicons of all six regions were cloned and a single clone was sequenced. Additionally, a more in-depth analysis was performed on region 4 and 5 by sequencing another 23 and 21 clones, respectively, because of the more informative nature of those regions regarding the differentiation between wild and vaccine type LSDV and the public availability of sequence information of other Capx isolates/strains. Following BlastN analysis of the obtained sequence of regions 1, 2 and 3 revealed a 99.9% (1 single nucleotide polymorphism (SNP) difference; region 3) to 100% (regions 1 and 2) identity with the GTPV strain Sudan (GenBank accession number MN072624) while there were 8, 21, 22 nucleotide (nt) differences with the closest LSDV, respectively. The obtained sequence of region 6, however, showed the highest similarity with LSDV (99.4%; 7 SNPs) while this was only 96.9% (37 nucleotide differences) with the GTPV Sudan. The difference between wild and vaccine type LSDV is too small in those 4 regions for further differentiation with a high confidence level. When looking at region 4, 14 out of the 24 clones (58%) were again similar to GTPV strain Sudan (99.4%; 0 to 4 nt differences). A second cluster of sequences (7 out of 24; 29.2%) aligned most closely to LSDV whereby 57% showed highest similarity to Neethling-LSD vaccine-OBP (accession number KX764645 [50]; 100% to 99.7%; 0 to maximal 2 nt differences) while 43% with wild type LSDV isolate Evros/GR/15 (accession number: KY829023 [51]; 100% to 99.9%; 0 to 1 nt difference). Three clones showed initially a higher sequence divergence but upon closer inspection of the alignment, it could be noted that these were hybrids in nature. One clone was similar to GTPV approximately up to base 428 after which the clone had a higher identity with LSDV. A second clone was similar but shifted at base 220 to LSDV. The third clone is exactly the inverse as it had first a higher identity with LSDV until base 440 and then with GTPV. This observation was confirmed by the RDP4 program as 5, 4 and 6 methods identified a potential recombination event in these three clones, respectively, between LSDV and GTPV. For region 5, all 22 clones were most closely related to LSDV. When wild type LSDV sequences were compared to vaccine sequences in that region, six informative SNPs could be noted. Twelve clones (54.5%) had 5 or 6 of those SNPs that were identical to the vaccine type LSDV while there were only 4 clones with this score for wild type LSDV. Six clones had an intermediate score. Some of these six clones had clusters of 3 and 4 wild type or vaccine type SNPs at one end of the fragment, which suggests possible hybrids; however, this could not be confirmed by RDP4.

### 3.3. Additional Characterization of OBP-Vac

Similar to the approach taken with Lumpivax, regions 1, 2, 4, 5 and 6 of the OBP-vac were cloned, sequenced and aligned. Due to limited source material and the less informative nature compared to the other regions, it was decided not to include region 3 in this analysis. In order to evaluate the potential presence of GTPV or wild type LSDV sequences multiple clones (between 20 and 25) were analyzed for each of the five regions. Vaccine LSDV was exclusively detected in all regions and the obtained clone sequences showed high identity with Neethling-based vaccine sequence present in GenBank for OBP (KX764645) [50]. In 52% to 80% of the clone sequences an 100% identity was found. Interestingly this was considerably less for region 2 as only 20% of the clones were 100% identical. Overall, the highest number of nucleotide differences observed in a clone sequence with the OBP GenBank sequence KX764645 was three, but the majority of them was 1.

### 3.4. Evaluation of Potential Generating Hybrid Fragments by PCR

Using the Cp-values obtained with the panCapx real-time PCR, the viral genome(s) present in the OBP and Caprivac vaccines were mixed in equimolar concentrations. Following amplification and cloning, 18 clones from region 4 were analyzed. By comparison of both parent sequences, 22 informative sites (SNP, indel) were identified (Supplementary Figure S1) that could be used to distinguish between LSDV and GTPV. Nine clones (50%) had OBP-like sequences (maximal 1 nt difference) while there were 7 GTPV Gorgan-like sequences (39%). In addition to these “parent-type” sequences, two clones showed a hybrid nature. One clone starts out like a GTPV-like sequence but changes to LSDV at the end with a break position between site 314 and 354. The second clone is the exact inverse as the start of the fragment is LSDV-like while the end is identical to GTPV with an apparent identical break area. The recombinant nature of this clone was confirmed by the RDP4 software as 6 out of 7 methods marked this clone as a potential recombinant.

In a similar approach, the fragments obtained with DIVA PCR-3 were cloned and 10 randomly selected clones were sequenced. No evidence of hybrids were found as the clone sequences were either almost identical (max 1 nt difference) to the OBP or the Caprivac parent.

### 3.5. In Vivo Evaluation of Lumpivax as a Vaccine Candidate

#### 3.5.1. Clinical Observations and Scoring

**Figure 1:**
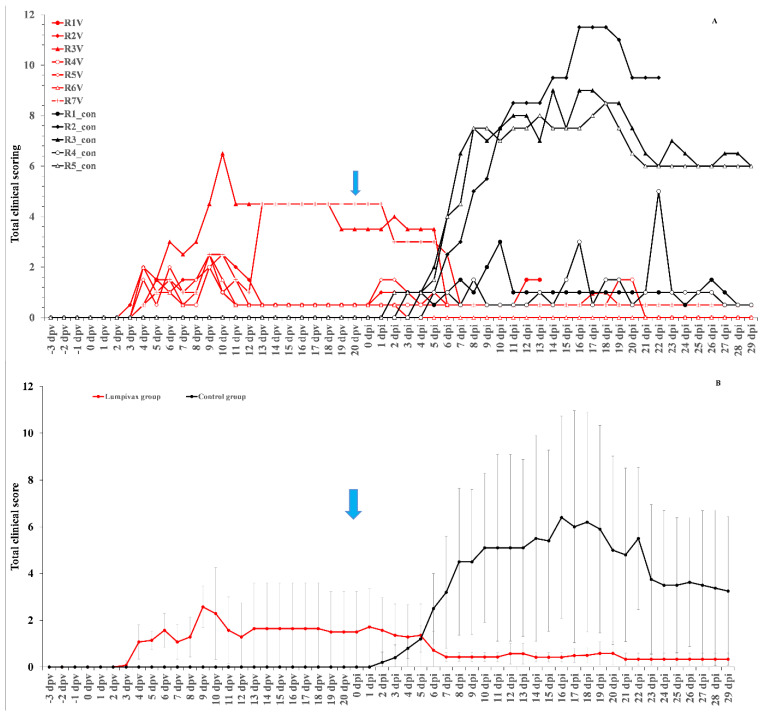
Total clinical of the Lumpivax and control group as individual scores (**A**) or as group average (**B**) whereby the variation is shown as error bars. Blue arrow: moment of challenge.

After injection with the Lumpivax vaccine, a raise in body temperature was seen in all animals (*n* = 7) as early as 3 dpv, with a peak between 7–9 dpv. Body temperatures returned back to normal between 10 and 14 dpv. This was accompanied by a reduced food uptake in all animals around 9 and 10 dpv. However, no impact was observed on general behavior/health status. Enlarged prescapular lymph nodes were observed in all animals, starting from 3 or 4 dpv, and remained enlarged until challenge. A clear local reaction was seen at the site of vaccination for all animals. Four animals had a moderate swelling and three animals (43%) had a severe swelling (>10 cm diameter) remaining visible throughout the animal trial. A Neethling response, characterized by generalized small nodule-like structures, appeared on 2 animals and this on 6 dpv and 13 dpv. They remained visible until 26/27 dpv. The individual total clinical scores of all control animals are displayed in Figure 1A.

After the inoculation with a virulent LSDV, all animals in the control groups (*n* = 5) developed a fever which spiked around 8 dpi. The highest body temperature measured was 40.6 °C, with all animals having a maximum body temperature above or equal to 40 °C. Following the fever spike, the body temperature returned relatively quickly (3 days) to normal in 20% of the control animals. In contrast, in 80% of the animals, the fever or elevated body temperatures remained for a prolonged period of time (>10 days). A reduced food uptake was seen in all control animals. A light (11 dpi) to severe (16 dpi) change in its general health status was observed only in one control animal and was therefore euthanized for ethical reasons at 22 dpi. Enlarged prescapular lymph nodes were observed in 60% of the animals (3/5) starting from 8 dpi onwards and remained enlarged until the end of the trial. The enlargement coincided with the observed fever spike and the development of nodules. Nodules appeared in 3 out of 5 animals (60%) between 6 and 8 dpi and remained visible until the end of the trial. The appearance of the permanent nodules began either localized (on 1 or 2 places on the animals, 100%) followed by a generalization after 1 or 2 days. Interestingly, all animals with permanent skin lesions had a prolonged fever while this was not observed in animals without skin lesions. The individual total clinical scores of all vaccinated animals is displayed in Figure 1A.

In the vaccinated group, a small fever response was only seen for one animal at 5 dpi, with a temperature of 39.4 °C, following the challenge with a virulent LSDV strain. All animals were clinically protected against other clinical signs, including the typical LSD nodule formation. The average total clinical scoring post-challenge of the vaccinated animals was substantially lower than that of the control animals (Figure 1B).

#### 3.5.2. Virology

No vaccine related viremia was observed in 4 out of 7 vaccinated animals (58%) with one additional animal being positive on a single sampling (9 dpv). Animal R7V, on the other hand, was positive on two consecutive samples at 7 and 9 dpv albeit with borderline Cp values (>40). A clear vaccine related viremia was seen for animal R3V as positive samples were obtained between 7 and 16 dpv with decreasing Cp-values. In order to identify the nature of the Capx present in those samples, the EDTA blood sample taken at 14 dpv (Cp of 37.3 on the panCapx real-time PCR) was analyzed with the DIVA-1, -2 ad -3. The DIVA-1 and -2 gave contradictory results. While the DIVA-1 typed the LSDV genome present in the sample as vaccine-like, the DIVA-2 revealed only wild type LSDV. The Cp values were in both cases approximately 37. A LSDV-like band was observed with DIVA PCR-3 after gel electrophoreses and was subsequently cloned. Sequence analysis of 18 clones showed a homogenous population of sequences (0 to max 3 nt differences) with the highest identity to the LSDV vaccine OBP (100 to 99%) while this was only 96% with wild type isolates like Evros (accession number: KY829023; 12 nt differences) [51]. At the moment of challenge, the blood samples of all vaccinated animals were negative and remained so thereafter until the end of the trial. As some of the animals displayed a Neethling response after vaccination, several biopsies (from nodules but also from skin with a normal outlook) and wound crusts were taken/collected from R3V (*n* = 4), R6V (*n* = 1) and R7V (*n* = 7) between 10 and 17 dpv. An additional buccal swab at 17 dpv was taken as well from R7V. Using the real-time DIVA PCR-1 and -2, two groups of sample results could be observed with one outlier. In a first group a signal was obtained for wild type and vaccine type LSDV simultaneously in the same sample, suggesting the presence of both. In contrast, in the second group of sample results only one signal was seen but the resulting LSDV status was contradictory between DIVA-1 and -2. While DIVA-1 indicated the presence of only vaccine type LSDV, DIVA-2 showed only wild type LSDV. These 2 group of sample results were seen in both animals from which multiple samples were available. When dividing the samples between biopsy or nodule/wound crust (*n* = 5) and biopsies of normal looking skin (*n* = 6) no exclusive relation was seen with the sample results. However, the combined detection of wild and vaccine type with DIVA-1 was more often seen in normal looking skin (67%) while this was only 20% in biopsy of nodules/wound crusts. All the DIVA-1 and -2 results are summarized in Table 1. The status of each sample was not only confirmed by repeat testing, the DNA samples were also analyzed pure and as a 1/10 dilution. In addition, DIVA-1 was also carried out in a singleplex adaptation with identical results.

In addition to the real-time PCRs, a number of samples (*n* = 10) were also screened with DIVA PCR-3. In most cases a LSDV-like band could be observed after gel electrophoresis (80%). In two of the eight samples, a second SPPV/GTPV like band was seen as well. Interestingly, in the biopsy of the normal looking skin R7V at 16 dpv only a wild type SPPV/GTPV like band was noted. The LSDV-like band was excised and purified from four of the six samples, which displayed only a LSDV-like band and two SPPV/GTPV-like bands, were cut-out from both samples which had LSDV and SPPV/GTPV-like bands (Table 1). Direct sequence analyses on the purified LSDV-like bands identified them either as vaccine LSDV (*n* = 3) or as wild type LSDV (*n* = 1; R3V vaccination site) while both SPPV/GTPV-like bands were confirmed as being SPPV/GTPV. The conservation in that region was too high to differentiate between both. In order to explore in more depth, the composition of the LSDV-like band of the R3V vaccination site, the DIVA-3 was repeated 4-fold. The LSDV-like bands of the 4 PCR reactions were cloned after extraction. A number of clones (3 to 5) of each repeat was sequenced and compared. In all 4 repeats, wild type and vaccine type LSDV sequences were found whereby in each repeat the clones which had a wild type sequence were dominant (2- to 3-fold).

At necropsy, 24 to 26 organ/tissue samples were collected for the vaccinated/challenged animals. A limited number of the samples taken, were found to be positive (7 to 25% per animal) on the panCapx real-time PCR and all had borderline Cp values (>38). The sample that had the highest positivity rate (67%) was the “normal” skin with an average Cp of 39. The presence of wild type LSDV was confirmed in one of the positive organ/tissue samples with DIVA-2. The sole exceptions were samples collected from the vaccination site (*n* = 3). The latter still scored strongly positive on the panCapx with Cp values between 20 and 21. Typing of these samples with DIVA-1 and -2 showed the combined presence of wild and vaccine type LSDV.

Viremia was detected in the control group in 3 of the 5 animals (60%) after challenge. The onset was between 6 and 7 dpi and the animals remained positive until the end of the trial. There is a 100% correlation between the development of viremia and noduli. The two animals without nodules remained negative in terms of blood with the exception of one single positive result on 13 dpi in one of the animals.

#### 3.5.3. Serology and Cellular Immunology

**Figure 2:**
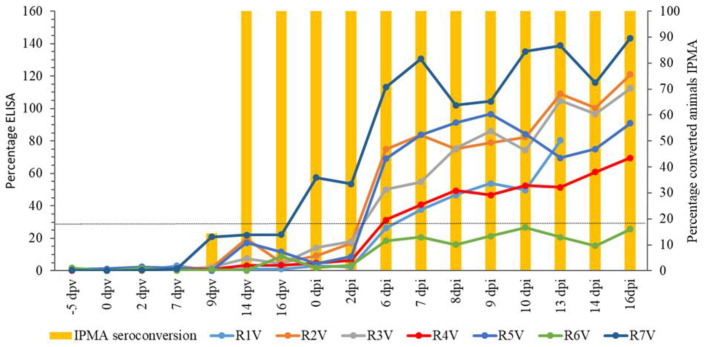
IPMA and ELISA results of the vaccinated group. Dotted line: positivity cut-off for ELISA.

The onset of seroconversion after vaccination was detected by IPMA (Figure 2) at 9 dpv (14.3%) while 100% seroconversion was seen at 14 dpv. Once seroconverted, all animals remained positive until the end of the trial. For ELISA (Figure 2), antibodies were not detected up to 16 dpv. At the day of challenge (21 dpv), one animal was positive for LSDV antibodies by ELISA. Five other animals became positive at 6 or 7 dpi. The earlier detection of LSDV antibodies with IPMA, compared to the ELISA, is in agreement with previous findings [39]. The animals that became positive during the trial (*n* = 6) stayed positive until the end of the trial. R6V remained negative during the trial in ELISA.

Analysis of the stimulated heparinized blood of the vaccinated animals showed an overall medium responsiveness. In total, 57% of the vaccination animals (4 out of 7) reacted during the post-vaccination phase and all of them did so with a medium intensity. At the moment of challenge, all animals scored negative again.

## 4. Discussion

Although many vaccination success stories can be found in veterinary medicine, it has also encountered opposition. Possible factors that play a role in vaccination hesitance by farmers are vaccine failure and (perceived or fear for) negative past experiences, such as side effects [52,53]. These negative factors can be due to properties of the vaccine itself, such as inadequate attenuation [54], and need to be addressed by vaccine safety studies; vaccine related issues can also be linked to the quality of the production/storage process. For the latter, an independent quality control (QC) procedure can provide help to demonstrate the quality of the finished product. When this is combined with an efficient communication of the results to the users it can increase the willingness to vaccinate [53]. During the vaccine virus identity control process, as essential part of the QC process, it can be useful to obtain additional genomic information without the necessity of complete genome sequencing especially for viruses with a long or segmented genome. For this purpose 4 additional gel-based PCRs were designed amplifying regions that are spread across the Capx genome. These PCRs were evaluated via an in silico study on publicly available Capx sequences and via successfully testing a number of SPPV, GTPV and LSDV including some vaccine strains (Supplementary Table S3). The cycling conditions were kept identical, allowing these PCRs to be combined which makes the process easier and faster. The amplicon size of the 4 PCRs is around 700 bp providing sufficient sequence information and permitting significant overlap when sequenced from both sides. This in turn enhanced the sequence quality in this study.

During the initial phase of the quality process of the Lumpivax vaccine anomalous results were observed when the DIVA PCRs were applied. Aside the expected LSDV vaccine signals, LSDV wild type and GTPV-like signals were simultaneously obtained and subsequently confirmed by sequencing. The presence of all three type of sequences were further confirmed with the phylogenetic PCRs. In this study the Taq polymerase was used in the phylogenetic PCR and DIVA PCR-3. This enzyme has an error rate between 2 × 10^−4^ [55] and 2 × 10^−5^ [56] and the necessary caution must be paid in the interpretation of unique SNPs [57]. This is especially true when this is combined with sanger sequencing. The number of nucleotide differences between LSDV and GTPV in the fragments analyzed in this study is at least 6 to 8 nucleotides (DIVA PCR-3, region) but is in general > 20 (region 2 to 6). Combined with the fact that the fragments were sequenced from both the ends with an overlap of 100% (region 1 to 5 and DIVA PCR-3), it is not possible that the finding of GTPV-like sequences is caused by PCR errors during amplification and sequencing. This holds also true for the finding of wild and vaccine type LSDV sequences although the nucleotide differences between them is less pronounced in the fragments (2/3 nt differences in region 1 and 2 but 6 to 10 in the other regions). That the observations are not caused by PCR errors is confirmed by repeat analysis of the DIVA-3 fragment as the LSDV wild and vaccine type differentiating SNPs were found in the clones of each repeat. The fact that no wild type LSDV and GTPV sequences were observed during the additional characterization of the OBP-vac, where even more clones were screened, shows that the observations done for the Lumpivax vaccine are not related to the methodology followed. The sequences found for OBP were homogenously linked to the Neethling vaccine strain with only unique SNPs present among the sequences. It is likely that a number of these unique SNPs are caused by PCR errors but the high frequency found in region 2, combined with the similarity among the fragments in size and composition (similar GC% pattern; data not shown), suggests that at least a number of them are true SNPs. This demonstrates the presence of minor Neethling variants in the vaccine.

The finding of the GTPV and LSDV wild and vaccine type sequences raises concerns about what is really present in the Lumpivax vaccine vial. Are all three viruses present in the vaccine or is (are) the vaccine virus(es) a recombinant(s) or is it a combination of both? Although limited, the current available data on Capx genomes seem to suggest a high degree of conservation through time. An almost complete conservation was seen in p32 gene of Indian GTPV isolates collected in 1946, 2004, and 2017 [58]. However, as this analysis is only a part of the genome whereby the exact selective pressure is unknown on that part of the genome, a certain degree of caution is always warranted when making generalized conclusions. However, the fact that the complete genome of the Bulgarian 2016 LSDV isolate shared a 99.99% nucleotide identity with an 2012 LSDV field isolates from Israel [59] confirms the low mutation rate. Aside acquiring mutations, insertions or deletions, genetic evolution is also driven by recombination events. The occurrence of such recombinations have already been established for a long time in co-cultures of poxviridae, such as vaccinia virus [60], or even between different poxviruses [61] and even in natural poxviridae co-infected animals [62]. Although the first tentative data about recombination events in Capx viruses were reported by Gershon et al. in 1988 [63], by means of restriction enzyme maps, no additional evidence emerged until recently. Vaccine-like LSDV recombinants were reported in Russia [64], where they were linked to outbreaks in 2017 [65] and 2019 [66] and in China [67]. Therefore the finding of LSDV-GTPV recombinant-like sequences in region 4 and to a minor extent in region 5 of the Lumpivax vaccine is intriguing as this suggests that recombinants are present in the vaccine vial. However, such recombinant-like fragments can be generated artificially with PCR by mixing different LDVs and GTPV DNAs together, as was shown in this study with OBP and Caprivac. On the other hand, no GTPV-like sequences were found in region 5, although multiple clones were screened. That this is caused by a low concentration of GTPV in the vaccine vial is unlikely as its presence was found in regions 1 to 3 in the single clone sequenced. Furthermore, in region 4, 58% of the clones were GTPV-like, suggesting that GTPV is at least co-dominant if the detections were caused by the virus itself. Although region 5 was successfully used on other GTPV strains, it cannot be 100% excluded that the lack of GTPV-like sequences is caused by not recognizing the unknown GTPV possibly present in the vaccine vial.

In order to investigate the impact on the efficacy of the vaccine and the diagnostics, the Lumpivax vaccine was used in an animal vaccination/challenge trial. Notwithstanding the presence of LSDV and GTPV-like sequences in the vaccine, the vaccine protected the vaccinated animals against a virulent LSDV challenge as no clinical signs were seen after challenge. The degree of protection, seroconversion and IFNg responsiveness were similar to other LSDV attenuated vaccines such as the OBP-vac used in this study [16] and only traces of Capx genome were found in the organs/tissues collected at necropsy. A Neethling response was observed after vaccination in a number of animals as this was also observed with some other LSDV LAVs [16]. Vaccine-related viremia was at least confirmed in one animal. Interestingly, when one of the Capx positive blood samples after vaccination was further characterized, a contradiction in LSDV status was seen between DIVA-1 and -3 on the one hand and DIVA-2 on the other hand. The first two PCRs typed the sample as LSDV vaccine while DIVA-2 indicated the presence of wild type LSDV. This contradiction was similarly seen in a number of biopsies that were taken around the same time as the positive blood sample. This is different from the in vitro results of the vaccine vial itself as there LSDV wild and vaccine type were always detected together by DIVA-1 and -2. The exact reason for this is currently unclear and warrants further study. As DIVA-1 is a duplex, a shift in ratio between wild and vaccine type LSDV could result in one of the two no longer being detected [33]. For this purpose DIVA-1 was also carried out as a singleplex. As this gave the same results the former explanation can be eliminated. Technician error and the presence of inhibitory elements can also been excluded as repeat and sample dilution testing always gave the same results. Therefore, the results could be explained by the presence of recombinants.

Aside from LSDV, GTPV-like sequences were also detected by DIVA-3, and confirmed by sequencing, in biopsies taken from three animals around 14/16 dpv. The detection of viral genome originating from the injected vaccine itself might be possible and has been demonstrated in the past after vaccination against bluetongue [68] but these detections were with real-time PCR and even then with borderline Cp’s. It is questionable that such very weak concentration could be detected with the gel-based PCR used in this study. In addition in one of the three animals the Capx genome was never detected in the blood during the trial and in a second animal only borderline positive results (panCapx real-time PCR; Cp > 41) were detected between 7 and 9 dpv. This makes it even more unlikely that the GTPV-like sequences came from the injected vaccine itself. If the hypothesis is followed whereby a GTVP strain is present in the vaccine, then this would mean that it multiplied in the host to a certain extent as it was detected in biopsies taken. It has been observed that some of the sheeppox and goatpox strains can cross infect their respective hosts to some degree [69,70]. However there is currently no data showing that GTPVs can multiply in cattle making this hypothesis less likely and increases the likelihood of the possible presence of recombinants in the Lumpivax vaccine.

The potential presence of recombinants has not only implications for the diagnostics but also for LSDV epidemiology. The problematic impact of the emergence of LSDV recombinants was shown by Byadovskaya et al. [71]. With the potential presence of recombinants in the blood and biopsies, the risk of transmission by vectors needs to be taken seriously. Stomoxys has been shown to transmit LSDV relatively easily [72] and LSDV vaccine-like sequences have been detected in Musca Domestica flies in Russia in 2017 [73] and in China in 2019 [74]. Recombinants may influence LSDV transmission also at another level. Indirect non-vectored transmission has always been considered to be of minor importance [75] but recent experiments with a recombinant LSDV strain demonstrated the possibility of contact transmission [76]. Whether this contact transmission was solely due to the recombinant nature of the used LSDV challenge strain needs further investigation.

## 5. Conclusions

The characterization of the vaccine virus present in the Lumpivax vaccine clearly demonstrates the necessity of an independent quality control policy as LSDV-wild type and GTPV-like sequences were found in this vaccine during the initial screening although this vaccine comes with a quality certificate. Similar findings were absent when characterizing the vaccine viruses in OBP-vac and Caprivac. This raises serious concerns about the content of Lumpivax and the potential presence of LSDV recombinants. Notwithstanding the fact that the vaccine proved to be protective against a LSDV challenge, the use of the vaccine could lead to a misinterpretation of the DIVA PCR results if only one DIVA PCR was performed. When the results of the additional characterization of the vaccine itself are combined with the results from the samples of the animal trial, the presence of LSDV recombinants is more likely to explain the observed results than the presence of different Capx strains in the vaccine. Further confirmation of our findings by whole genome sequencing would be advisable. Whether the recombinant nature of the LSDV vaccine strain has an influence on the potential for transmission needs further investigation but the fact that recombinant LSDV strains are found recently in the field is worrying. Finally, the Capripox heterogeneity observed in the vaccine is troublesome as it is unclear how this will vary, batch to batch, and evolve with the creation of new or additional recombinations. Therefore, quality control of each new batch is warranted.

## Figures and Tables

**Table 1:** PCR results of the biopsies, wound crusts and swab taken after vaccination. (a): All biopsies and wound crusts were collected at least 10 cm away of the vaccination site, except the biopsy of the vaccination site itself; NT: not tested; WT: wild type LSDV signal; VAC: vaccine type LSDV signal; WT + VAC: a wild type and vaccine type LSDV signal; *: was excised for direct sequencing.

Time	Sample (a)	panCapx	DIVA-1	DIVA-2	DIVA-3
10 dpv	R3V biopsy vaccination site	15.97	WT + VAC	WT + VAC	LSDV *
14 dpv	R3V biopsy nodule	35.00	VAC	WT	SPPV/GTPV * + LSDV
14 dpv	R3V biopsy normal looking skin 1	NT	VAC	WT + VAC	LSDV
16 dpv	R3V biopsy normal looking skin 2	36.83	WT + VAC	WT + VAC	no signal
14 dpv	R6V biopsy nodule	32.45	VAC	Neg	SPPV/GTPV * + LSDV
14 dpv	R7V biopsy nodule	30.2	WT + VAC	WT + VAC	LSDV
14 dpv	R7V wound crust 1	19.69	VAC	WT	LSDV *
14 dpv	R7V wound crust 2	19.61	VAC	WT	LSDV *
14 dpv	R7V Biopsy normal looking skin	NT	WT + VAC	NT	LSDV *
16 dpv	R7V biopsy normal looking skin 1	18.10	VAC	WT	SPPV/GTPV
16 dpv	R7V biopsy normal looking skin 2	35.29	WT + VAC	WT + VAC	no signal
16 dpv	R7V Swab Nasal	30.76	WT + VAC	Neg	no signal
17 dpv	R7V biopsy normal looking skin	31,22	WT + VAC	WT + VAC	no signal

## Data Availability

The main data presented in this study are available within the study itself and other data may be made available through contact with the corresponding author.

## Supplementary Materials

The following are available online at https://www.mdpi.com/article/10.3390/vaccines9091019/s1, Figure S1: Alignment of region 4 of both parent (OBP and Caprivac) and 2 hybrid-like clone sequences, Table S1: Additional information of the phylogenetic PCRs used; (a): ORFs based upon Tulman et al. 2002 [1], Table S2: List of Capripox viruses/vaccines used to study potential recombination in region 4 clones of the lumpivax vaccine, Table S3: List of Capripox viruses/vaccines used to validate the new phylogenetic PCRs, Table S3: List of Capripox viruses/vaccines used to validate the new phylogenetic PCRs.

## References

[1] Tulman E.R., Afonso C.L., Lu Z., Zsak L., Sur J.H., Sandybaev N.T., Kerembekova U.Z., Zaitsev V.L., Kutish G.F., Rock D.L. (2002). The genomes of sheeppox and goatpox viruses. J. Virol..

[2] Tuppurainen E., Alexandrov T., Beltrán-Alcrudo D. (2017). Lumpy Skin Disease Field Manual—A Manual for Veterinarians.

[3] Gupta T., Patial V., Bali D., Angaria S., Sharma M., Chahota R. (2020). A review: Lumpy skin disease and its emergence in India. Vet. Res. Commun..

[4] FAO (2020). Introduction and Spread of Lumpy Skin Disease in South, East and Southeast Asia—Qualitative Risk Assessment and Management.

[5] Alemayehu G., Zewde G., Admassu B. (2013). Risk assessments of lumpy skin diseases in Borena bull market chain and its implication for livelihoods and international trade. Trop. Anim. Health Prod..

[6] Gari G., Bonnet P., Roger F., Waret-Szkuta A. (2011). Epidemiological aspects and financial impact of lumpy skin disease in Ethiopia. Prev. Vet. Med..

[7] Green H.F. (1959). Lumpy Skin Disease: Its Effect on Hides and Leather and a Comparison on this Respect with some other Skin Diseases. Bull. Epizoot. Dis. Afr..

[8] Babiuk S., Bowden T.R., Boyle D.B., Wallace D.B., Kitching R.P. (2008). Capripoxviruses: An emerging worldwide threat to sheep, goats and cattle. Transbound. Emerg. Dis..

[9] Tageldin M.H., Wallace D.B., Gerdes G.H., Putterill J.F., Greyling R.R., Phosiwa M.N., Al Busaidy R.M., Al Ismaaily S.I. (2014). Lumpy skin disease of cattle: An emerging problem in the Sultanate of Oman. Trop. Anim. Health Prod..

[10] Abera Z., Degefu H., Gari G., Ayana Z. (2015). Review on Epidemiology and Economic Importance of Lumpy Skin Disease. Int. J. Basic Appl. Virol..

[11] Ramaswamy N. (1994). Draught Animals and Welfare. Rev. Sci. Technol..

[12] Molla W., de Jong M.C.M., Gari G., Frankena K. (2017). Economic Impact of Lumpy Skin Disease and Cost Effectiveness of Vaccination for the Control of Outbreaks in Ethiopia. Prev. Vet. Med..

[13] Ben-Gera J., Klement E., Khinich E., Stram Y., Shpigel N.Y. (2015). Comparison of the Efficacy of Neethling Lumpy Skin Disease Virus and x10RM65 Sheep-Pox Live Attenuated Vaccines for the Prevention of Lumpy Skin Disease—The Results of a Randomized Controlled Field Study. Vaccine.

[14] Tuppurainen E.S.M., Venter E.H., Shisler J.L., Gari G., Mekonnen G.A., Juleff N., Lyons N.A., De Clercq K., Upton C., Bowden T.R. (2017). Review: Capripoxvirus Diseases: Current Status and Opportunities for Control. Transbound. Emerg. Dis..

[15] Klement E., Broglia A., Antoniou S.E., Tsiamadis V., Plevraki E., Petrović T., Polaček V., Debeljak Z., Miteva A., Alexandrov T. (2020). Neethling Vaccine Proved Highly Effective in Controlling Lumpy Skin Disease Epidemics in the Balkans. Prev. Vet. Med..

[16] Haegeman A., De Leeuw I., Mostin L., Campe W.V., Aerts L., Venter E., Tuppurainen E., Saegerman C., De Clercq K. (2021). Comparative Evaluation of Lumpy Skin Disease Virus-Based Live Attenuated Vaccines. Vaccines.

[17] Abutarbush S.M., Hananeh W.M., Ramadan W., Al Sheyab O.M., Alnajjar A.R., Al Zoubi I.G., Knowles N.J., BachanekBankowska K., Tuppurainen E.S. (2016). Adverse Reactions to Field Vaccination Against Lumpy Skin Disease in Jordan. Transbound. Emerg. Dis..

[18] Hamdi J., Boumart Z., Daouam S., El Arkam A., Bamouh Z., Jazouli M., Tadlaoui K.O., Fihri O.F., Gavrilov B., El Harrak M. (2020). Development and Evaluation of an Inactivated Lumpy Skin Disease Vaccine for Cattle. Vet. Microbiol..

[19] Wolff J., Moritz T., Schlottau K., Hoffmann D., Beer M., Hoffmann B. (2020). Development of a Safe and Highly Efficient Inactivated Vaccine Candidate against Lumpy Skin Disease Virus. Vaccines.

[20] Nims R., Price P.J. (2017). Best practices for detecting and mitigating the risk of cell culture contaminants. In Vitro Cell. Dev. Biol. Anim..

[21] Lucey B.P., Nelson-Rees W.A., Hutchins G.M. (2009). Henrietta, Lacks, HeLa cells, and cell culture contamination. Arch. Pathol. Lab. Med..

[22] Mirjalili A., Parmoor E., Moradi Bidhendi S., Sarkari B. (2005). Microbial contamination of cell cultures: A 2 years study. Biologicals.

[23] Barone P.W., Wiebe M.E., Leung J.C., Hussein I.T.M., Keumurian F.J., Bouressa J., Brussel A., Chen D., Chong M., Dehghani H. (2020). Viral contamination in biologic manufacture and implications for emerging therapies. Nat. Biotechnol..

[24] Fox K.A., Kopanke J.H., Lee J.S., Wolfe L.L., Pabilonia K.L., Mayo C.E. (2019). Bovine viral diarrhea in captive Rocky Mountain bighorn sheep associated with administration of a contaminated modified-live bluetongue virus vaccine. J. Vet. Diagn. Investig..

[25] Su Q., Liu X., Li Y., Meng F., Cui Z., Chang S., Zhao P. (2019). The intracorporal interaction of fowl adenovirus type 4 and LaSota strain significantly aggravates the pathogenicity of one another after using contaminated Newcastle disease virus-attenuated vaccine. Poult. Sci..

[26] Bumbarov V., Golender N., Erster O., Khinich Y. (2016). Detection and isolation of Bluetongue virus from commercial vaccine batches. Vaccine.

[27] Savini G., Lorusso A., Paladini C., Migliaccio P., Di Gennaro A., Di Provvido A., Scacchia M., Monaco F. (2014). Bluetongue serotype 2 and 9 modified live vaccine viruses as causative agents of abortion in livestock: A retrospective analysis in Italy. Transbound. Emerg. Dis..

[28] Sangula A.K., Siegismund H.R., Belsham G.J., Balinda S.N., Masembe C., Muwanika V.B. (2011). Low diversity of foot-and-mouth disease serotype C virus in Kenya: Evidence for probable vaccine strain re-introductions in the field. Epidemiol. Infect..

[29] World Organization for Animal Health (OIE) (2019). Manual of Diagnostic Tests and Vaccines for Terrestrial Animals, Chapter 3.4.12: Lumpy Skin Disease. https://www.oie.int/fileadmin/Home/eng/Health_standards/tahm/3.04.12_LSD.pdf.

[30] Obernier J.A., Baldwin R. (2006). Establishing an appropriate period of acclimatization following transportation of laboratory animals. ILAR J..

[31] Vandenbussche F., Vandemeulebroucke E., De Clercq K. (2010). Simultaneous detection of bluetongue virus RNA, internal control GAPDH mRNA, and external control synthetic RNA by multiplex real-time PCR. Methods Mol. Biol..

[32] Haegeman A., Zro K., Vandenbussche F., Demeestere L., Van Campe W., Ennaji M.M., De Clercq K. (2013). Development and validation of three Capripoxvirus real-time PCRs for parallel testing. J. Virol. Methods.

[33] Agianniotaki E.I., Chaintoutis S.C., Haegeman A., Tasioudi K.E., De Leeuw I., Katsoulos P.D., Sachpatzidis A., De Clercq K., Alexandropoulos T., Polizopoulou Z.S. (2017). Development and validation of a TaqMan probe-based real-time PCR method for the differentiation of wild type lumpy skin disease virus from vaccine virus strains. J. Virol. Methods.

[34] Vidanović D., Tešović B., Šekler M., Debeljak Z., Vasković N., Matović K., Koltsov A., Krstevski K., Petrović T., De Leeuw I. (2021). Validation of TaqMan-Based Assays for Specific Detection and Differentiation of Wild-Type and Neethling Vaccine Strains of LSDV. Microorganisms.

[35] Chibssa T.R., Grabherr R., Loitsch A., Settypalli T.B.K., Tuppurainen E., Nwankpa N., Tounkara K., Madani H., Omani A., Diop M. (2018). A gel-based PCR method to differentiate sheeppox virus field isolates from vaccine strains. Virol. J..

[36] Lamien C.E., Le Goff C., Silber R., Wallace D.B., Gulyaz V., Tuppurainen E., Madani H., Caufour P., Adam T., El Harrak M. (2011). Use of the Capripoxvirus homologue of Vaccinia virus 30 kDa RNA polymerase subunit (RPO30) gene as a novel diagnostic and genotyping target: Development of a classical PCR method to differentiate Goat poxvirus from Sheep poxvirus. Vet. Microbiol..

[37] Haegeman A., Zro K., Sammin D., Vandenbussche F., Ennaji M.M., De Clercq K. (2016). Investigation of a Possible Link Between Vaccination and the 2010 Sheep Pox Epizootic in Morocco. Transbound. Emerg. Dis..

[38] Rozen S., Skaletsky H. (2000). Primer3 on the WWW for general users and for biologist programmers. Methods Mol. Biol..

[39] Haegeman A., De Leeuw I., Mostin L., Van Campe W., Aerts L., Vastag M., De Clercq K. (2020). An Immunoperoxidase Monolayer Assay (IPMA) for the detection of lumpy skin disease antibodies. J. Virol. Methods.

[40] Nicholas K., Nicholas H., Deerfield D.W. (1997). GeneDoc: A tool for editing and annotating multiple sequence alignments. Embnet. News.

[41] Martin D.P., Murrell B., Golden M., Khoosal A., Muhire B. (2015). RDP4: Detection and analysis of recombination patterns in virus genomes. Virus Evol..

[42] Martin D., Rybicki E. (2000). RDP: Detection of recombination amongst aligned sequences. Bioinformatics.

[43] Padidam M., Sawyer S., Fauquet C.M. (1999). Possible emergence of new geminiviruses by frequent recombination. Virology.

[44] Martin D.P., Posada D., Crandall K.A., Williamson C. (2005). A modified bootscan algorithm for automated identification of recombinant sequences and recombination breakpoints. AIDS Res. Hum. Retrovir..

[45] Lam H.M., Ratmann O., Boni M.F. (2018). Improved Algorithmic Complexity for the 3SEQ Recombination Detection Algorithm. Mol. Biol. Evol..

[46] Posada D., Crandall K.A. (2001). Evaluation of methods for detecting recombination from DNA sequences: Computer simulations. Proc. Natl. Acad. Sci. USA.

[47] Gibbs M.J., Armstrong J.S., Gibbs A.J. (2000). Sister-scanning: A Monte Carlo procedure for assessing signals in recombinant sequences. Bioinformatics.

[48] Smith J.M. (1992). Analyzing the mosaic structure of genes. J. Mol. Evol..

[49] Mathijs E., Vandenbussche F., Haegeman A., Al-Majali A., De Clercq K., Van Borm S. (2016). Complete Genome Sequence of the Goatpox Virus Strain Gorgan Obtained Directly from a Commercial Live Attenuated Vaccine. Genome Announc..

[50] Mathijs E., Vandenbussche F., Haegeman A., King A., Nthangeni B., Potgieter C., Maartens L., Van Borm S., De Clercq K. (2016). Complete Genome Sequences of the Neethling-Like Lumpy Skin Disease Virus Strains Obtained Directly from Three Commercial Live Attenuated Vaccines. Genome Announc..

[51] Agianniotaki E.I., Mathijs E., Vandenbussche F., Tasioudi K.E., Haegeman A., Iliadou P., Chaintoutis S.C., Dovas C.I., Van Borm S., Chondrokouki E.D. (2017). Complete Genome Sequence of the Lumpy Skin Disease Virus Isolated from the First Reported Case in Greece in 2015. Genome Announc..

[52] Gethmann J., Zilow V., Probst C., Elbers A.R., Conraths F.J. (2015). Why German farmers have their animals vaccinated against Bluetongue virus serotype 8: Results of a questionnaire survey. Vaccine.

[53] Wane A., Dione M., Wieland B., Rich K.M., Yena A.S., Fall A. (2020). Willingness to Vaccinate (WTV) and Willingness to Pay (WTP) for Vaccination Against Peste des Petits Ruminants (PPR) in Mali. Front. Vet. Sci..

[54] Tuppurainen E.S., Pearson C.R., Bachanek-Bankowska K., Knowles N.J., Amareen S., Frost L., Henstock M.R., Lamien C.E., Diallo A., Mertens P.P. (2014). Characterization of sheep pox virus vaccine for cattle against lumpy skin disease virus. Antivir. Res..

[55] Saiki R.K., Gelfand D.H., Stoffel S., Scharf S.J., Higuchi R., Horn G.T., Mullis K.B., Erlich H.A. (1988). Primer-directed enzymatic amplification of DNA with a thermostable DNA polymerase. Science.

[56] McInerney P., Adams P., Hadi M.Z. (2014). Error Rate Comparison during Polymerase Chain Reaction by DNA Polymerase. Mol. Biol. Int..

[57] Bracho M.A., Moya A., Barrio E. (1998). Contribution of Taq polymerase-induced errors to the estimation of RNA virus diversity. J. Gen. Virol..

[58] Roy P., Jaisree S., Balakrishnan S., Senthilkumar K., Mahaprabhu R., Mishra A., Maity B., Ghosh T.K. (2018). Karmakar, A.P. Molecular epidemiology of goat pox viruses. Transbound. Emerg. Dis..

[59] Mathijs E., Vandenbussche F., Ivanova E., Haegeman A., Aerts L., De Leeuw I., Van Borm S., De Clercq K. (2020). Complete Coding Sequence of a Lumpy Skin Disease Virus from an Outbreak in Bulgaria in 2016. Microbiol. Resour. Announc..

[60] Qin L., Evans D.H. (2014). Genome scale patterns of recombination between coinfecting vaccinia viruses. J. Virol..

[61] Bedson H.S., Dumbell K.R. (1964). Hybrids derived from the viruses of variola major and cowpox. J. Hyg..

[62] Strayer D.S., Skaletsky E., Cabirac G.F., Sharp P.A., Corbeil L.B., Sell S., Leibowitz J.L. (1983). Malignant rabbit fibroma virus causes secondary immunosuppression in rabbits. J. Immunol..

[63] Gershon P.D., Kitching R.P., Hammond J.M., Black D.N. (1989). Poxvirus genetic recombination during natural virus transmission. J. Gen. Virol..

[64] Sprygin A., Pestova Y., Bjadovskaya O., Prutnikov P., Zinyakov N., Kononova S., Ruchnova O., Lozovoy D., Chvala I., Kononov A. (2020). Evidence of recombination of vaccine strains of lumpy skin disease virus with field strains, causing disease. PLoS ONE.

[65] Sprygin A., Babin Y., Pestova Y., Kononova S., Wallace D.B., Van Schalkwyk A., Byadovskaya O., Diev V., Lozovoy D., Kononov A. (2018). Analysis and insights into recombination signals in lumpy skin disease virus recovered in the field. PLoS ONE.

[66] Sprygin A., Van Schalkwyk A., Shumilova I., Nesterov A., Kononova S., Prutnikov P., Byadovskaya O., Kononov A. (2020). Full-length genome characterization of a novel recombinant vaccine-like lumpy skin disease virus strain detected during the climatic winter in Russia, 2019. Arch. Virol..

[67] Lu G., Xie J., Luo J., Shao R., Jia K., Li S. (2021). Lumpy skin disease outbreaks in China, since 3 August 2019. Transbound. Emerg. Dis..

[68] De Leeuw I., Garigliany M., Bertels G., Willems T., Desmecht D., De Clercq K. (2015). Bluetongue virus RNA detection by real-time rt-PCR in post-vaccination samples from cattle. Transbound. Emerg. Dis..

[69] Babiuk S., Bowden T.R., Parkyn G., Dalman B., Hoa D.M., Long N.T., Vu P.P., Bieu do X., Copps J., Boyle D.B. (2009). Yemen and Vietnam capripoxviruses demonstrate a distinct host preference for goats compared with sheep. J. Gen. Virol..

[70] Gelaye E., Belay A., Ayelet G., Jenberie S., Yami M., Loitsch A., Tuppurainen E., Grabherr R., Diallo A., Lamien C.E. (2015). Capripox disease in Ethiopia: Genetic differences between field isolates and vaccine strain, and implications for vaccination failure. Antivir. Res..

[71] Byadovskaya O., Pestova Y., Kononov A., Shumilova I., Kononova S., Nesterov A., Babiuk S., Sprygin A. (2020). Performance of the currently available DIVA real-time PCR assays in classical and recombinant lumpy skin disease viruses. Transbound. Emerg. Dis..

[72] Sohier C., Haegeman A., Mostin L., De Leeuw I., Campe W.V., De Vleeschauwer A., Tuppurainen E.S.M., van den Berg T., De Regge N., De Clercq K. (2019). Experimental evidence of mechanical lumpy skin disease virus transmission by Stomoxys calcitrans biting flies and Haematopota spp. horseflies. Sci. Rep..

[73] Sprygin A., Pestova Y., Prutnikov P., Kononov A. (2018). Detection of vaccine-like lumpy skin disease virus in cattle and Musca domestica L. flies in an outbreak of lumpy skin disease in Russia in 2017. Transbound. Emerg. Dis..

[74] Wang Y., Zhao L., Yang J., Shi M., Nie F., Liu S., Wang Z., Huang D., Wu H., Li D. (2021). Analysis of vaccine-like lumpy skin disease virus from flies near the western border of China. Transbound. Emerg. Dis..

[75] Weiss K.E. (1968). Lumpy skin disease virus. Virol. Monogr..

[76] Kononov A., Byadovskaya O., Wallace B.D., Prutnikov P., Pestova Y., Kononova S., Nesterov A., Rusaleev V., Lozovoy D., Sprygin A. (2020). Non-vector-borne transmission of lumpy skin disease virus. Sci. Rep..

