# Supplementary material for: The Importance of Quality Control of LSDV Live Attenuated Vaccines for Its Safe Application in the Field
Source: Vaccines (Basel). 2021 Sep 13;9(9):1019. doi: 10.3390/vaccines9091019 (PMC8472990; doi:10.3390/vaccines9091019)
Supplement: Supplementary file 1 [file vaccines-09-01019-s001.zip › Figure S1.pdf]

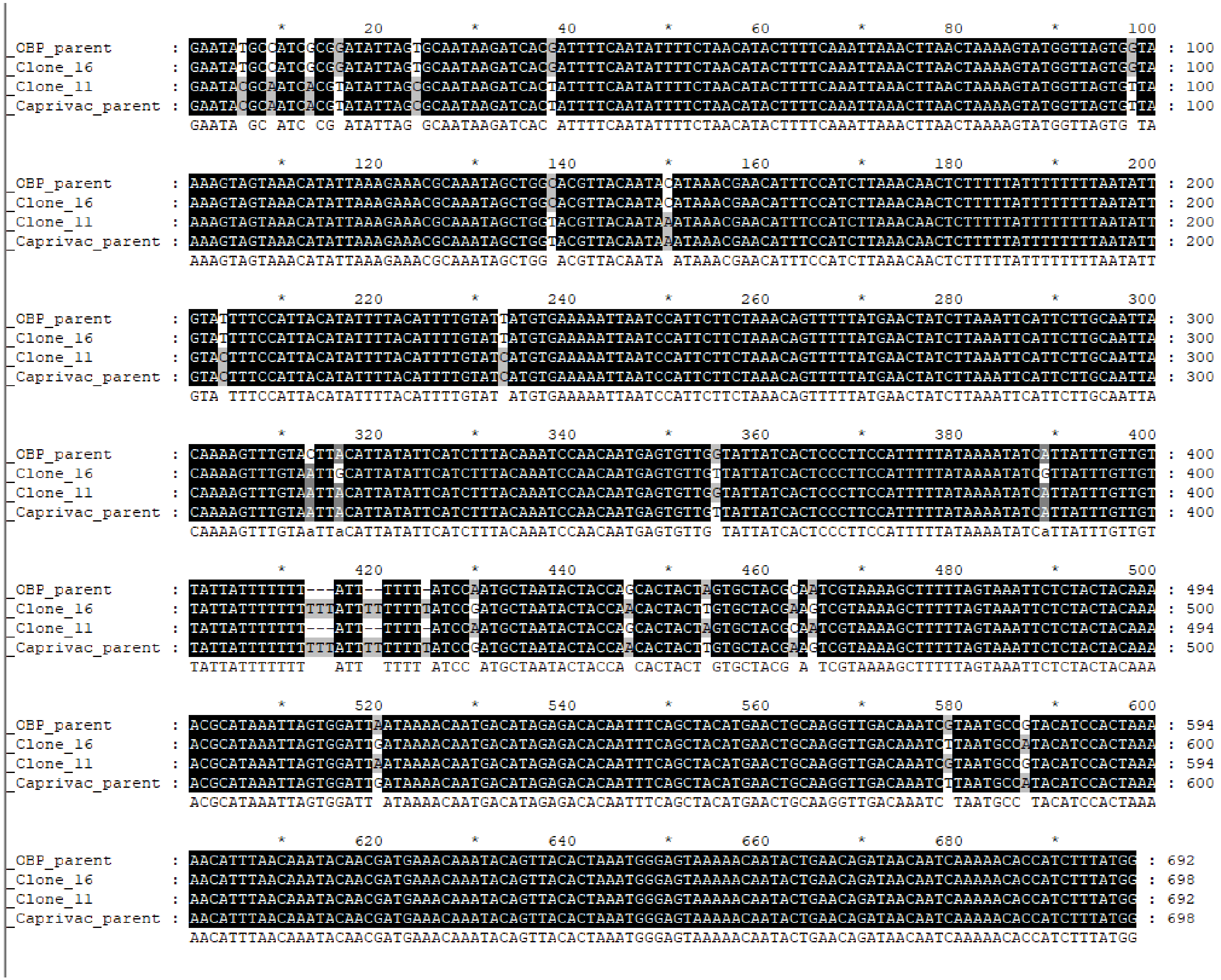

Figure S1: Alignment of region 4 of both parent (OBP and Caprivac) and 2 hybrid-like clone sequences
