# Supplementary material for: The Importance of Quality Control of LSDV Live Attenuated Vaccines for Its Safe Application in the Field
Source: Vaccines (Basel). 2021 Sep 13;9(9):1019. doi: 10.3390/vaccines9091019 (PMC8472990; doi:10.3390/vaccines9091019)
Supplement: Supplementary file 1 [file vaccines-09-01019-s001.zip › Table S1.pdf]

Referred in text as	Targeted region (a)	Primer sequence (F: Forward / R: Reverse)	Amplicon length	Reference
**Published PCRs**				
Region 6	RPO30	F: ATTCGTTATCGCAGAACAAGG / R: CACCAACCATAGAATAGTATTGAGAC	1188	35
Region 5	ORF 25/26	F: TTCGTTTTCAGCGATTTTATTT / R: AGGAGATTTTATTATGAGTGGCTTT	735	36
**Newly developed PCRs**				
Region 1	ORF83	F: GAGAAACCGCAACAGGAAAA / R: GGATGAGCAACGAACCAACT	614	
Region 2	ORF116/117	F: TGGAGAAATGGAAAGGGATTG / R: CAGGCGACGATGATGAAAC	750	
Region 3	ORF58/59	F: TTTTATGGCGTTCCACGATT / R: CCCAACACTCTCTCGCTTCA	755	
Region 4	ORF10	F: ACCCAACAACACAAGGAAGG / R: CATCGCAAACAAAGAATAAGAAAG	708	

Table S1: Additional information of the phylogenetic PCRs used; (a): ORFs based upon Tulman et al. 2002 [1]
