# Supplementary material for: The Importance of Quality Control of LSDV Live Attenuated Vaccines for Its Safe Application in the Field
Source: Vaccines (Basel). 2021 Sep 13;9(9):1019. doi: 10.3390/vaccines9091019 (PMC8472990; doi:10.3390/vaccines9091019)
Supplement: Supplementary file 1 [file vaccines-09-01019-s001.zip › Table S2.pdf]

Sheeppox strains/isolates/vaccines	Accession Number
PoxDoll vaccine	in-house
Jovac Vaccine	in-house
Moroccan vaccine	in-house
Romania vaccine	in-house
Field Isolate Morocco 2010	in-house
Arbel	in-house
**Lumpy skin disease strains/isolates/vaccines**	**Accession Number**
Herbivac vaccine	KX764644
Neethling vaccine LW1959	AF409138
SIS Lumpyvax_vaccine	KX764643
KSGP vaccine 0240	KX683219
Evros GR15	KY829023
LSDV field isolate Isreal	in-house
Serbia Bujanovac 2016	KY702007
**Goatpox strains/isolates**	**Accession Number**
India	MN072620
Vietnam	MN072621
Oman	MN072623
Gorgan	in-house
FZ	KC951854
Yemen	MN072625
Sudan	MN072624

Table S2: List of capripox viruses / vaccines used to study potential recombination in region 4 clones of the lumpivax vaccine.
