# Supplementary material for: The Importance of Quality Control of LSDV Live Attenuated Vaccines for Its Safe Application in the Field
Source: Vaccines (Basel). 2021 Sep 13;9(9):1019. doi: 10.3390/vaccines9091019 (PMC8472990; doi:10.3390/vaccines9091019)
Supplement: Supplementary file 1 [file vaccines-09-01019-s001.zip › Table S3.pdf]

Sheeppox isolates / strains/ vaccines
10 Field isolates from Morocco 2010
vaccine from Morocco
Kenya
Pakistan
Arbel
Nigeria
Romanian
Turkey
SPPV PoxDoll vaccine
SPPV Jovac vaccine
**Sheep-Goat isolates / strains**
Kano
Isiolo
Kedong
Yemen
Sudan
**Lumpy skin disease isolates / strains**
LSDV2
LSDV Bovivax vaccine
LSDV OBP vaccine
LSDV Lumpyvax vaccine
LSDV NDOLL vaccine
LSDV Herbivac vaccine
LSDV Lumpyvac vaccine
LSDV field isolate Cyprus from 2015
**Goatpox isolates / strains/ vaccines**
Gorgan
India
Bangladesh

Table S3: List of Capripox viruses / vaccines used to validate the new phylogenetic PCRs.
